# Anatomical Modularity of Verbal Working Memory? Functional Anatomical Evidence from a Famous Patient with Short-Term Memory Deficits

**DOI:** 10.3389/fnhum.2017.00231

**Published:** 2017-05-17

**Authors:** Eraldo Paulesu, Tim Shallice, Laura Danelli, Maurizio Sberna, Richard S. J. Frackowiak, Chris D. Frith

**Affiliations:** ^1^Psychology Department and Milan Centre for Neuroscience, University of Milano-BicoccaMilan, Italy; ^2^fMRI Unit, IRCCS Istituto Ortopedico GaleazziMilan, Italy; ^3^Institute of Cognitive Neuroscience, University College LondonLondon, United Kingdom; ^4^Cognitive Neuroscience Sector, SISSA, International School for Advanced StudiesTrieste, Italy; ^5^Department of Diagnostic Neuroradiology, Niguarda Ca' Granda HospitalMilan, Italy; ^6^Department of Clinical Neurosciences, University Hospital of LausanneLausanne, Switzerland; ^7^Ecole Polytechnique Fédérale de Lausanne, BioTech CampusGeneva, Switzerland; ^8^Wellcome Trust Centre for Neuroimaging, Institute of Neurology, University College LondonLondon, United Kingdom; ^9^Institute of Philosophy, School of Advanced Studies, University of LondonLondon, United Kingdom

**Keywords:** working memory, phonological loop, neuro-reductionism, recovery from aphasia, brain activation, PET, verbal short-term memory, fMRI

## Abstract

Cognitive skills are the emergent property of distributed neural networks. The distributed nature of these networks does not necessarily imply a lack of specialization of the individual brain structures involved. However, it remains questionable whether discrete aspects of high-level behavior might be the result of localized brain activity of individual nodes within such networks. The phonological loop of working memory, with its simplicity, seems ideally suited for testing this possibility. Central to the development of the phonological loop model has been the description of patients with focal lesions and specific deficits. As much as the detailed description of their behavior has served to refine the phonological loop model, a classical anatomoclinical correlation approach with such cases falls short in telling whether the observed behavior is based on the functions of a neural system resembling that seen in normal subjects challenged with phonological loop tasks or whether different systems have taken over. This is a crucial issue for the cross correlation of normal cognition, normal physiology, and cognitive neuropsychology. Here we describe the functional anatomical patterns of JB, a historical patient originally described by Warrington et al. (1971), a patient with a left temporo-parietal lesion and selective short phonological store deficit. JB was studied with the H_2_^15^O PET activation technique during a rhyming task, which primarily depends on the rehearsal system of the phonological loop. No residual function was observed in the left temporo-parietal junction, a region previously associated with the phonological buffer of working memory. However, Broca's area, the major counterpart of the rehearsal system, was the major site of activation during the rhyming task. Specific and autonomous activation of Broca's area in the absence of afferent inputs from the other major anatomical component of the phonological loop shows that a certain degree of functional independence or modularity exists in this distributed anatomical-cognitive system.

## Introduction

Working memory (WM) is one of the most studied domains of human mental faculties. Behavioral investigations in normal subjects have contributed to the development of articulated models such as those stemming from the initial model of Baddeley and Hitch (1974). These models also found some support from anatomical observations in brain damaged patients and functional anatomical studies in normal subjects (see Dolan et al., 1997; Muller and Knight, 2006; Buchsbaum and D'Esposito, 2008 for reviews).

Central to the assumptions of current models of WM is the concept that a certain degree of modularity exists within the system. The comparison of normal and pathological behavioral patterns has supported this notion at the functional cognitive level (Shallice and Vallar, 1990; Repovs and Baddeley, 2006). However, demonstration of such modularity or at least of some degree of functional independence of the underlying neural systems has turned out not to be an obvious task, primarily because of the methodological limitations of individual methods taken *per se* (for a discussion, see Paulesu et al., 1996a; Henson, 2005).

We previously argued that either functional imaging in normal subjects or neuropsychological methods alone cannot provide unequivocal support for this assumption, rather a combination of such methods may be necessary (Paulesu et al., 1996a; Shallice and Cooper, 2012). This paper presents an attempt at testing some of the assumptions of the most widely supported WM model, that of Baddeley and Hitch (1974) using a combination of functional anatomical and neuropsychological techniques.

Among the systems of the Baddeley and Hitch working memory model, the verbal slave system—the phonological loop, focus of the experimental data reported in this paper—seemed best suited to our aims, because of its simplicity and relatively well-understood architecture at the cognitive level.

The phonological loop allows maintenance of verbal material through active rehearsal, for example, when trying to remember a telephone number (Murray, 1968; Levy, 1971). In Salamé and Baddeley's version (1982), the phonological loop has two components: a [input] short-term phonological store (STMS), based on a phonological code (Salamé and Baddeley, 1982), and a subvocal rehearsal process, based on an articulatory code (Baddeley et al., 1975).

Independence of these components is indicated by the fact that differential interference of concurrent articulation (articulatory suppression) on the word length (Baddeley et al., 1975) and phonological similarity effects. The word length effect refers to the fact that it is harder to retain words that take longer to articulate in working memory (e.g., **harpoon, Friday, coerce** as opposed to **bucket, wiggle, tipple**; Baddeley et al., 1975). The phonological similarity effect refers to the difficulty in remembering words that sound similar (***can***, ***mad, sat*** as opposed to ***bed, hall, frost***) (Conrad, 1964). Articulatory suppression abolishes the word length effect (Baddeley et al., 1975), but not the phonological similarity effect (Murray, 1968; Levy, 1971), when stimuli are presented aurally. This finding indicates that the rehearsal process is based on a high-level articulatory code that is independent of short-term storage to which auditory-verbal stimuli have privileged and direct—that is, not mediated by rehearsal-access. However, when study material is presented visually, articulatory suppression also abolishes the phonological similarity effect indicating that visual material needs rehearsal, or phonological recoding, prior to retention in a short-term phonological input store (Murray, 1968). Articulatory suppression, but not unattended speech (Burani et al., 1991), has a small detrimental effect on various kinds of phonological awareness tasks, such as rhyming tasks, stress assignment tasks, homophony tasks for pseudo-words when stimuli are presented visually (Wilding and White, 1985; Besner, 1987; Burani et al., 1991). These results suggested that the rehearsal process tends to be used in phonological awareness tasks, while the contribution of the STMS, which is the site of interference by unattended speech (Salamé and Baddeley, 1982), is considered marginal when short-term memory demands of the phonological awareness task are small (Burani et al., 1991).

### Short-term memory patients

Certain aspects of the normal multi-component cognitive model of the phonological loop were initially obtained by inference from observations in brain damaged patients with selective impairment of verbal short-term memory (Warrington and Shallice, 1969; Warrington et al., 1971; Vallar and Baddeley, 1984; Shallice and Vallar, 1990; Waters et al., 1992; Vallar et al., 1997). The behavioral deficit of some of these patients has been interpreted as the result of damage to a short-term phonological buffer (Shallice and Butterworth, 1977; see also Shallice and Vallar, 1990 for a review of these cases), while other patients have patterns of performance more consistent with an impairment of the rehearsal process (Waters et al., 1992; Vallar et al., 1997).

The study of the lesion pattern of short-term memory (STM) patients has also contributed converging evidence on the multi-component nature of verbal working memory. Meta-analysis of brain lesions in patients with deficits of the STMS suggested that a crucial lesion site may be in the left inferior parietal cortex in the perisylvian region (Warrington et al., 1971; Shallice and Vallar, 1990; Vallar et al., 1997; Baldo and Dronkers, 2006) while there is now some evidence that patients with a dysfunctional rehearsal process tend to have lesions in Broca's area (Vallar et al., 1997) or in the left insula (Dronkers, 1996)^1^.

Experiments on virtual lesions such as those provoked by TMS or direct cortical stimulation during awake surgery support the overall picture, Thus, Romero et al. (2006) were able to determine short-term memory deficits after TMS inhibition over the key regions of the phonological loop (Broca's area and the left temporo-parietal junction). Moreover, Papagno et al. (2017) have recently shown that electrical inhibition of the left supramarginal gyrus is associated with predominant order errors in auditory span task, a characteristic deficit in phonological input buffer patients, and, in particular, in JB in whom they were frequent (Shallice and Butterworth, 1977).

Taken together, the lesion data provide converging evidence with the growing body of functional imaging literature of verbal working memory which points to a multi-component normal neural architecture of the phonological loop in which Broca's area is the major counterpart of the rehearsal system while the left temporo-parietal junction operates as a phonological buffer (see for example, Paulesu et al. (1993) and the more detailed literature review in the discussion of the present paper).

### Unaddressed issues in STM patients studies and motivations for the present study

As much as there is a reinsuring consistency between psychological and neuropsychological findings for the domain of verbal short-term memory, there is one aspect that remains unaddressed to date: *the consistency between the normal and the pathological model at a functional anatomical level*, that is, the consistency of the functional anatomical operations of the normal phonological loop and the operations of its remains in specific patients. As discussed earlier in this introduction, one such exploration may have a more general interest as it allows one to test one of the basic assumptions of cognitive neuropsychology, namely that the mind and its neural underpinnings shows some degree of modularity and that modules can be damaged in a fairly selective manner while other components are left relatively untouched.

Indeed, to make an effective inference from neuropsychological findings to models of normal function along the aforementioned lines one has to exclude the possibility that residual behavioral abilities in a brain damaged patient do not arise from a re-organization of the relevant cognitive-anatomical system. For language-related functions such as phonological short-term memory, one might expect a priori that preserved abilities are being subserved either by the activity of remaining parts of the left hemisphere, if reorganization has not occurred, or are taken over by the right hemisphere. The latter possibility makes the linking of normal and abnormal cognitive anatomical models more complex.

Lesion studies based on structural imaging techniques are insufficient to investigate these possibilities because they lack any functional information about brain regions spared by damage. However, access to functional imaging techniques and the availability of **patient JB** (Warrington et al., 1971; Shallice and Butterworth, 1977; Shallice, 1988; Butterworth et al., 1990), has allowed us to translate into practical experimental questions the general issue raised in this introduction: do the residual phonological skills of such patients arise from preserved brain areas that are normally active in phonological tasks? Do these areas operate as expected by normal cognitive-anatomical models, or do the patients' preserved abilities arise from the operation of different parts of a reorganized brain?

There is a second reason for exploring the functional localization of JB's verbal working memory system in more detail. Buchsbaum and D'Esposito (2008) have written a major critique of the concept of the phonological input buffer on the basis of functional imaging and neuropsychological evidence (see also Buchsbaum et al., 2011). They produced an alternative perspective in which they argue that there is a perceptual-motor speech interface system, which, when damaged, produces a severe impairment in phonological short-term memory, while leaving speech perception and speech production relatively less affected. This component is held to be in the area SPT (sylvian parietal temporal) lying at the junction of the temporal and parietal lobes in the posterior part of the auditory association cortex (part of area TPT in Galaburda and Sanides, 1980 terminology). JB is the putative phonological input buffer patient most extensively discussed in this critique. It is therefore appropriate to put on record what we dicovered of the functional anatomy of the remains of her verbal working memory system.

Interestingly, the consistency of functional anatomical patterns of brain damaged patients with WM disorders and the normal patterns has not been assessed as yet.

## Methods

### Case report and rationale for the study in patient JB

JB a right-handed secretary born in 1935, is a patient with very limited verbal span (2–3 items) despite normal speech comprehension, speech output, and intelligence (for a summary of her performance on a number of specific tasks, see Table 1; Warrington et al., 1971; Shallice and Butterworth, 1977). Her verbal span capacity has remained as such until the time of our PET experiment. At the age of 24, the removal of a meningioma in the left parietal region led to a lesion in the left temporo-parietal cortex. She was initially severely aphasic but she recovered very satisfactorily, except for a pronounced verbal short-term memory deficit. Her normal performance on verbal long-term memory tasks has supported findings in another patient, KF (Warrington and Shallice, 1969), showing that the two verbal memory systems are dissociable (Warrington et al., 1971). As with other patients with a similar pattern of symptoms (e.g., patient PV originally described by Basso et al., 1982), it has been postulated that JB's selective deficit of auditory verbal short-term memory is due to damage to a verbal short-term store which is based on a phonological code and which is a sub-component of the phonological loop of working memory (Warrington and Shallice, 1969; Shallice and Butterworth, 1977; Vallar and Baddeley, 1984; Shallice and Vallar, 1990). JB's performance on psychological testing did not change with time, until her eventual death, some time after she participated in this study and more than 40 years after her operation. More specifically, the other major part of the phonological loop, the rehearsal system based on an articulatory code (Baddeley et al., 1975; Salamé and Baddeley, 1982), appeared to be spared in JB as she was able to speak normally (Shallice and Butterworth, 1977) and to segment the sound of words for phonological discrimination (e.g., during rhyming tasks). JB's anatomical lesion, as defined on the basis of angiography (Warrington et al., 1971), was also consistent with evidence from normal subjects that the phonological buffer localizes to the left temporo-parietal junction (Paulesu et al., 1993; Demonet et al., 1994).

**Table 1:** **Basic Word Processing Performance of patient JB**.

**SPEECH AND LANGUAGE**
Speech production^1^: Normal, except for a slight increase in function word errors
Reading and spelling^2^: Normal
Minimal pair phoneme discrimination^3^: 95–99%
Newcastle speech segmentation (lesser)^3^: 94%
Sentence comprehension: Characteristic of STM patients^2^^,^^3^^,^^4^
Naming words from description^2^: 100%
Word reproduction^3^: 91% (no effect syllabic length; imageability; frequency)
**SPAN AND SHORT-TERM MEMORY TESTS**
Auditory verbal^2^: Digits, 3.4, Letters 2.5, Words 2.5
(Errors^2^: Order > Acoustic > Perseverative)
Visual verbal^2^: 90% on 4 digit strings; 70% on 4 letter strings
For meaningful sounds^5^: Normal
Auditory probe digit^5^: 37% errors on 6 digit lists
Word probe 12 item lists^3^: Excessive errors throughout list
Span-effect of phonological similarity^3^^,^^8^: Variable across experiments
Span-word length effect^3^^,^^8^: Not consistent
Recency effect in free recall^2^: Reduced to one item
Sternberg paradigm (Auditory 3 digits)^7^: 81%, ERP-P450 130 ms delayed
Sternberg paradigm (Visual 3 digits)^7^: 100%, ERP-P450 not delayed

Results taken from:

1 Shallice and Butterworth, 1977;

2 Warrington et al., 1971;

3 Howard and Shallice, unpublished;

4 Caplan and Waters, 1990;

5 Shallice and Warrington, 1970;

6 Shallice and Warrington, 1977;

7 Starr and Barrett, 1987;

8 *Shallice, unpublished. ERP, Event-related potential*.

It remained to be investigated, however, whether other anatomical components of the phonological loop that subserve rehearsal, primarily Broca's area, were spared and capable of activation when JB is challenged with phonological tasks which in normal subjects put minimal demands on short-term memory but involve sub-vocalization for rehearsal and similar processes (Vallar and Baddeley, 1984; Burani et al., 1991). If JB's preserved phonological loop skills depended on spared regions of the anatomical system described in normal subjects, then we would predict activation in those areas during phonological tasks. To test this prediction we used the 15O-water positron emission tomography (PET) technique to measure relative regional cerebral blood flow (rCBF) changes as an index of altered synaptic activity. We used a phonological task that normally activates Broca's area, left insula, superior temporal cortex and supplementary motor cortex, mesial ventral extrastriate cortex and cerebellum (Paulesu et al., 1993, 1996b).

A further non-trivial issue was the exact localization of brain damage in patient JB. This was previously mapped by inference on the basis of angiography (Warrington et al., 1971). It was held to be impossible to perform an MRI scan on her because of incomplete records about the presence of intracranial metallic clips, the operation having been carried out in 1959.

However, PET scanners themselves have sufficient spatial resolution to make precise anatomical assignations and the added advantage that these show distant functional effects due to brain damage (Feeney and Baron, 1986). We were therefore able to map explicitly the brain lesion^2^ of this paradigmatic patient using PET data (see PET methods section).

The study was approved by the Hammersmith Hospital Medical Ethics Committee and permission to administer radioactivity was obtained from the ARSAC, UK. JB's consent was obtained according to the declaration of Helsinki.

### Experimental design

The experiment was designed to test the functioning of what was left of the phonological loop of patient JB. Of course, testing JB with a full blown short-term memory task would have made little sense as the patient had a severe limitation of the verbal span due to her phonological buffer deficit. We therefore employed the procedure of using a task that makes demands on the process which would be relatively minor for a normal subject but activates the rest of the rehearsal system normally. We know that STM patients when doing the easier task of single letter matching, given by auditory input, perform more slowly than normal subjects (Starr and Barrett, 1987). So it seems plausible that the phonological STM load that this task involves, which is minor in normal subjects, is considerable for STM patients. Performance of a rhyming task for two items clearly requires that, while the matching process is carried out, the phonological representations of the new letter name and the target stimulus/b/ are held in STM with a memory load close to JB's span limit.

**Figure 1:**
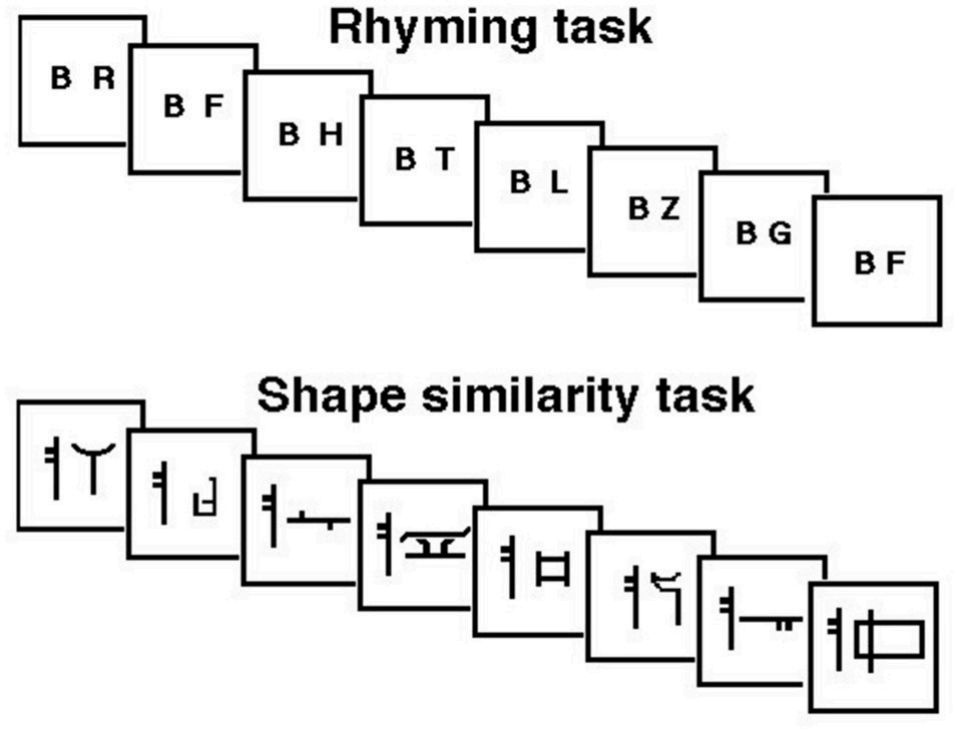
**Schematic representation of the rhyming task for letter names and its control task (shape similarity judgments for Korean letters) phonological similarity (rhyming) task: subjects were asked to make rhyme judgments about consonants appearing on a computer screen at a rate of one per second**. They moved a joy-stick toward a “yes” symbol every time a letter appeared that rhymed with the letter “B” which was always present on the screen. Rhyming letters occurred at a frequency of 1 in 6. Shape similarity task: subjects were asked to judge whether a false font looked similar to a target false letter always present on the screen.

JB was therefore tested with a continuous rhyming task using letter names that were presented visually—to guarantee that the rehearsal system was involved—as in Paulesu et al. (1993). She was asked to detect letter names rhyming with “B” (e.g., C, D, G, etc.). The letter “B” was always present on the screen. Targets occurred at random at a rate of 1 in 6. Brain activity measured during the rhyming task was compared to that measured during a control task, a shape similarity judgment task for simple line drawings modified from the Korean alphabet (see Figure 1). This task controlled for visual stimulation and cognitive components (e.g., immediate matching to sample) thus isolating letter recognition and phonological processing. JB performed both tasks silently six times in a counterbalanced order. She raised her right first finger to indicate the detection of a target and did not speak during scanning.

### PET methods

#### Data acquisition and pre-processing

The distribution of rCBF was measured by recording radioactivity tomographically following the intravenous injection of ^15^O-labeled water (H_2_^15^O) with the CTI 953B PET scanner (CTI Inc., Knoxville, TN, USA).

Twelve consecutive regional blood flow (rCBF) measurements (six for each experimental and control condition) with PET were collected. Each rCBF scan was divided into two frames: (i) 30-s measurement of the background radiation; (ii) 2.45-min rCBF measurement with concurrent psychological stimulation.

Data were acquired by scanning in 3D mode (Townsend et al., 1991). ${\text{H}}_{2}^{15}$O was infused (10 ml min^−1^; 55 MBq ml^−1^) as a tracer of blood flow while scans were acquired. After attenuation correction (measured by a transmission scan), the data were reconstructed as 31 transaxial planes by three-dimensional filtered back projection with a Hanning filter of cut-off frequency 0.5 cycles voxel^−1.^ The resolution of the resulting images was 8.5 × 8.5 × 4.3 mm at full width half-maximum (FWHM) (Spinks et al., 1992). The integrated counts accumulated were used as an index of rCBF (Mazziotta et al., 1985; Fox and Mintun, 1989).

All PET scans were realigned to the first one by using an automated algorithm (Woods et al., 1992). On an average rCBF image we then used the stereotactic normalization procedures of SPM^3^: the parameters estimated for the average rCBF image were then applied to the individual PET scans. The images were then smoothed with a 16 × 16 × 16 gaussian filter.

Lesion mapping. This was done by comparing JB's average rCBF distribution across all scans with the average rCBF images of 12 normal controls who took part in PET studies on the phonological loop and were scanned under similar conditions, for six scans of phonological memory or rhyming; and for six scans of visual feature discrimination/memory for false fonts. The statistical comparison was made by using a two-sample *t*-test, after normalization for global counts (statistical threshold *p* < 0.001 with cluster level correction 0.05 FWE). The “lesion image” resulting from the statistical comparison between the patient and 12 controls was then mapped with reference to the stereotactic space of the Montreal Neurological Institute (MNI) using the Automatic Anatomical Labeling (AAL) template (Tzourio-Mazoyer et al., 2002) with the software MRICron (http://www.nitrc.org/projects/mricron).

#### Comparison of JB's lesion and the results of previous imaging experiments on phonological short-term memory

To test the degree of overlap of JB's functional lesion with previous imaging data on the phonological loop, the data of representative papers (Paulesu et al., 1993; Paulesu et al., this paper; Demonet et al., 1994; Awh et al., 1996; Salmon et al., 1996; Smith et al., 1996, 1995; Buchsbaum et al., 2005) reporting foci in the temporo-parietal junction and in the parietal cortex were submitted to a meta-analysis using the Activation Likelihood Estimate approach (Eickhoff et al., 2009). The ensuing clusters of significant convergence of regional effects were then overlapped with JB's lesion. The ALE analysis was thresholded at *p* < 0.001.

#### Analysis of activation data

Activations evoked by the rhyming task were assessed on a voxel by voxel basis using statistical parametric mapping. Global differences in CBF across scans were compensated for using proportional scaling and comparisons of means were made using the t statistic. The resulting set of t values, constituting a statistical parametric map (SPM{t}), was then transformed into a SPM{Z} map. The statistical threshold *p* < 0.001 was used for those areas that are known to be involved in the phonological loop in normal subjects (Paulesu et al., 1993). This threshold, takes into account the number of areas tested in a hypothesis led analysis (the areas of the phonological loop). For other areas a harsher threshold was used (*p* < 0.05 corrected for multiple non-independent comparisons).

All analyses were performed with SPM12 (http://www.fil.ion.ucl.ac.uk/spm/software/spm12).

All results are reported according to MNI strereotactic coordinates: for comparison of the new data with previous PET data (Paulesu et al., 1993, 1996b), the “older” data were converted from Talairach space into MNI coordinates according to technique described by M. Brett (http://imaging.mrc-cbu.cam.ac.uk/imaging/MniTalairach).

## Results

### Distribution of JB's brain lesion

**Figure 2:**
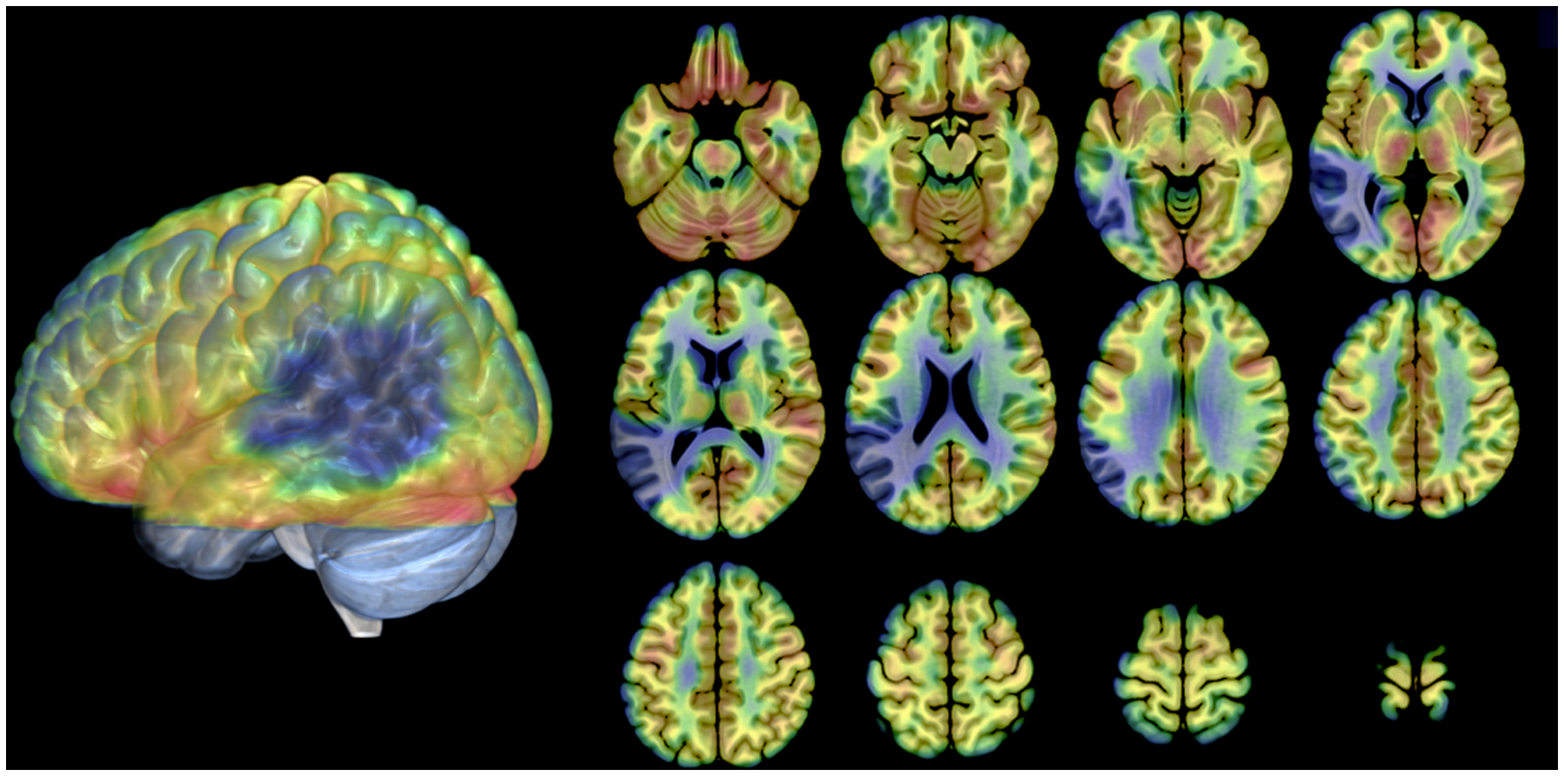
**Distribution of the anatomical lesion in patient JB**. The figure illustrates an average blood flow image of the patient from all scans after realignment and stereotactic normalization. The PET data have been superimposed on a normal MRI scan conforming to the same stereotactic space. Axial cuts and the lateral view of the 3D rendering are shown. The brain damage is indicated by low flow (blue areas). Gray areas in the 3D rendering: not covered by the PET scans.

Analysis of average rCBF distribution across the 12 PET scans showed the extent of the brain lesion: this involved the left inferior parietal lobule, the left angular gyrus, the left supramarginal gyrus, the left superior, middle and inferior temporal gyri, the left fusiform gyrus and the middle and inferior occipital gyrus (see Table 2 and Figure 2).

**Table 2:** **Lesion distribution in patient JB**.

**Brain region**	**Lesion volume (mm^3^)**	**Percentage of overall lesion**	**Stereotactic coordinates and *Z* scores of regional local maxima**			
			***x***	***y***	***z***	***Z* score**
Left inferior parietal	1,158	1.9	−56	−32	36	5.5
Left angular gyrus	6,266	10.3	−60	−56	24	6.2
			−56	−54	28	6.2
			−50	−70	−26	5.7
Left supramarginal gyrus	4,470	7.4	−60	−50	24	6.3
			−58	−54	26	6.3
			−66	−34	24	4.8
Left superior temporal gyrus	8,355	13.8	−62	−48	16	6.3
			−62	−50	20	6.3
Left middle temporal gyrus	22,615	37.3	−60	−50	18	6.3
			−58	−52	24	6.3
			−46	−54	12	6.2
Left inferior temporal gyrus	3,869	6.4	−48	−60	−4	5.5
Left middle occipital gyrus	9,406	15.5	−50	−72	16	6.1
			−46	−74	22	5.8
			−40	−60	0	5.0
Left inferior occipital gyrus	2,587	4.3	−50	−66	−2	5.5
Left fusiform gyrus	1,078	1.8	−50	−56	−8	4.8
			−46	−60	−14	4.1

*The anatomical mapping of the SPM hypo-perfusion -lesion- map was obtained with the software MRIcron (www.mricron.com) using the template AAL. On the right end side of each row, we report the stereotactic coordinates and Z scores of the region specific local maxima effects*.

**Figure 3:**
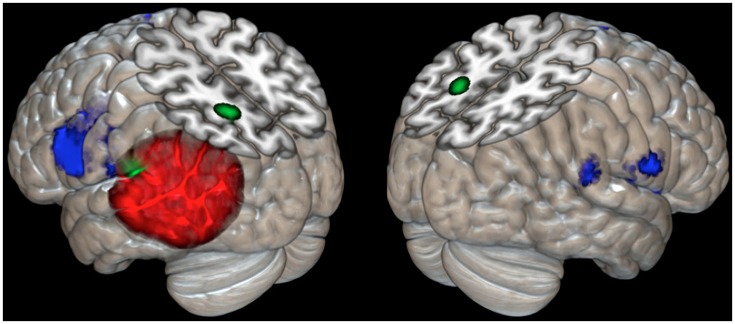
**Cortical rendering of the left and right brain areas activated in JB during a rhyming task (blue areas), of the localization of her cerebral lesion involving the left temporo-parietal regions (red left posterior area) and of the results of meta-analysis on eight studies (Paulesu et al., 1993; Paulesu et al., Supplementary Material in this paper; Demonet et al., 1994; Smith et al., 1995, 1996; Awh et al., 1996; Salmon et al., 1996; Buchsbaum et al., 2005) investigating the functional correlates of verbal working memory and phonological buffer (areas in green)**.

The meta-analysis of the imaging data on the phonological loop revealed two clusters: the first at the left temporo-parietal junction (*x* = −54; *y* = −31; *z* = 23 contributions from Paulesu et al., 1993, 1996b; Demonet et al., 1994; Salmon et al., 1996; Buchsbaum et al., 2005 from STM Sternberg paradigms and the like); a second and separate more posterior and dorsal clusters (*x* = −20; *y* = −61; *z* = 44 contributions from Awh et al., 1996; Smith et al., 1996 from n-back tasks). Of the two clusters only the former falls within the boundaries of JB's lesion (Figure 3).

### PET activation experiment

Normal controls in a previous experiment performed on average 98% correct for the rhyming task and 97% correct for the shape similarity task (Paulesu et al., 1996b).

JB's performance in both tasks was well-above chance (rhyming task: 77% correct; shape similarity judgment task: 98% correct). Her performance on the rhyming task showed improvement over the experiment, achieving 90% in the last two blocks. This result confirms that JB had relatively spared phonological abilities, despite her very limited verbal short-term memory.

The results of the comparison of rCBF distributions in the rhyming task with its baseline were clear-cut (see Table 3 and Figure 3) and remarkably similar to the results of a group of normal subjects challenged with the same task (see Table 3, coordinates in small bold print). The maximal activation, in terms of extent and statistical significance, was in the left inferior frontal gyrus.

**Table 3:** **Brain areas activated during the rhyming task in patient JB and in normal controls**.

	**Left hemisphere**				**Right hemisphere**			
	***x***	***y***	***z***	***Z* score**	***x***	***y***	***z***	***Z* score**
**BRAIN REGIONS**								
SMA	–	–	–	–	6	10	76	3.2
	–	–	–	–	2	14	48	3.1
	**−*14***	***5***	***61***	***3.7***	–	–	–	–
Lateral premotor cortex	−50	8	16	4.8	60	2	16	3.5
	−48	−4	38	4.1	64	−6	16	3.0
	−***38***	***5***	***35***	***7.4***				
Inferior frontal gyrus	−54	10	18	4.9	54	10	12	3.1
	−52	16	26	3.6	–	–	–	–
	−***46***	***12***	***14***	***6.5***	–	–	–	–
Rolandic operculum	−52	4	14	5.1	56	6	16	3.1
	−48	−22	24	3.3	–	–	–	–
Insula	−42	−8	10	3.4	48	8	−6	3.3
	–42	−2	10	3.3	40	14	−4	3.1
	−***34***	−***6***	−***5***	***3.3***	***34***	***12***	***5***	***3.7***
	−***38***	***14***	***1***	***3.8***	–	–	–	–
	−***36***	***32***	***15***	***6.0***	–	–	–	–
Superior temporal gyrus	^*^	^*^	^*^	^*^	62	−36	22	3.3
	−***46***	−***27***	***3***	***6.1***	–	–	–	–
	−***44***	−***34***	***11***	***3.9***	–	–	–	–
Lenticular nucleus	−22	0	14	3.3	–	–	–	–
Caudate nucleus	−20	6	20	3.2	–	–	–	–
	−***6***	***18***	***10***	***4.9***	***18***	−***13***	***4***	***4.5***
	−***22***	***22***	***14***	***2.9***	–	–	–	–
Cerebellum^+^	−***12***	−***59***	−***18***	***3.3***	***18***	−***65***	−***23***	***3.5***

Coordinates refer to the maximal activation indicated by the highest Z score in a particular cerebral structure. Stereotactic coordinates in bold italic print refer to the brain areas activated in a group of five normal subjects reported in Paulesu et al. (1996b) after transformation to MNI stereotactic space using Brett's procedure. Distances are relative to the anterior commissure in a brain volume oriented along the ac-pc line. SMA, Supplementary motor area.

* In patient JB, the mirror region of the left hemisphere at −62, −36, 22 was significantly hypo-perfused (p < 0.05 FWE corrected).

+ *, The cerebellum was sampled inconsistently in patient JB*.

As in normal subjects challenged with the rhyming task, activation was also observed in the left and right anterior insula, the head of the caudate nucleus and the SMA. JB also showed activation of the mirror region to Broca's area in the right hemisphere and in the right temporo-parietal junction.

## Discussion of the PET experiment in patient JB

The experiment shows that JB has a lesion which incorporates the most frequently damaged region in patients with phonological short-term memory deficits (Shallice and Vallar, 1990), while the *surviving brain tissue* of the phonological loop, Broca's area, was *normally perfused* and *activated*, just as in normal controls.

These clear-cut findings when seen in the context the simple and generally accepted phonological short-term memory model introduced by the Salamé and Baddeley (1982), allow us to address two general questions.

The first general question is whether some neuropsychological syndromes can be explained by a simple process of subtracting one or more components or connections from the whole system. Activation studies in pathological cases of acquired brain damage (and some developmental disorders, Demonet et al., 2004) are critical for this purpose.

The second question is whether cognitive processes above the level of sensory or perceptual ones can be mapped onto brain anatomy to localize specific functional subsystems. This is an important issue for all those cognitive functions that have no animal model.

Before entering into the details of our discussion it is important to emphasize that the imaging literature on normal verbal working memory has grown considerably since the times of the initial descriptions of Paulesu et al. (1993). A careful review of this literature and its compatibility with the phonological loop model would require a dedicated review article in its own right, something beyond the scope of this manuscript (see for example, Buchsbaum and D'Esposito, 2008). Accordingly, in what follows, we concentrate on the papers that were acquired with methods designed to highlight functional anatomical effects that would correlate, at a similar grain size^4^, with the phenomenology that is normally seen in patients with a phonological buffer deficit or in normal subjects during specific behavioral paradigms. We will also discuss the major challenges to the phonological loop functional anatomical model to date.

### Models of the phonological loop: normal models and anatomical lessons from patient JB

The multi-component nature of the phonological loop has also been supported by functional anatomical models in normal subjects of the mid 90s (Paulesu et al., 1993; Demonet et al., 1994; for discussions see Demonet et al., 1996; Paulesu et al., 1996a; Dolan et al., 1997). In our early PET activation experiment we showed that the phonological loop depends primarily on left peri-sylvian cortices and on brain regions involved in planning speech (Paulesu et al., 1993). By comparing the patterns of activation associated with a rhyming task, and those of a phonological short-term memory task, we observed that the left temporo-parietal junction was significantly more active in the short-term memory task, taking into account the patterns of activation in the control non-verbal tasks; therefore we suggested that the short-term phonological input store localizes to the left temporo-parietal junction^5^. The same area was significantly more active for the same comparison in a group of dyslexic subjects (Paulesu et al., unpublished observations, see Appendix in Supplementary Material) and in a group of healthy subjects during a non-word learning task (Paulesu et al., 2009). A very similar result emerged from Demonet et al.'s study (Demonet et al., 1994) in which a complex phonological awareness task was used with high phonological short-term memory demands and was contrasted with a semantic awareness task. The phonological task involved judging whether a given sound (e.g., /b/) occurred before another target sound (e.g., /d/): in the critical conditions a non-word stimulus like “rabudabu” was a target with a stimulus like “radubabu” as a foil. Importantly, this experiment involved auditory stimulation, and thus provided an across modality cross-validation of Paulesu et al.'s (1993) findings which used visually presented materials. One other replication of the same basic findings was published by Salmon et al. (1996) who used Paulesu et al.'s (1993) stimuli.

Even though interpretations with respect to general theoretical models vary (see Buchsbaum and D'Esposito, 2008 for contrasting views on the phonological buffer), these general functional anatomical findings have passed the challenge of replication: for example, in the work of Buchsbaum et al. (2005) the region they called SpT has sustained activity during short-term memory active maintenance for both visually and auditorily presented stimuli that are indistinguishable in stereotactic coordinates from those discussed above.

Now, let's consider the functional anatomy of JB's phonological loop and her skills. From Table 3, it can be seen that, with one proviso, the pattern of activation for the rhyming task is substantially identical to that of normal subjects, except that no activity is found in superior temporal cortex and the temporo-parietal junction where anatomical damage has occurred.

In agreement with a cognitive-physiological model that supposes a certain degree of modularity within the anatomy of the phonological loop (Paulesu et al., 1993; Demonet et al., 1996; Vallar et al., 1997), these imaging data show that a patient with a *well-placed* lesion has a disruption of the behavior attributed to the damaged *cognitive-anatomical module* (phonological store impairment by damage to left temporo-parietal cortex). This observation corroborates structural imaging investigations (Warrington et al., 1971; Shallice and Vallar, 1990; see also Vallar et al., 1997 for another case). The PET activation study, also shows that *preserved* phonological loop skills such as rehearsal, which involves higher levels of the speech production system, are associated with activity in spared *cognitive*-*anatomical systems* that in the case of the rehearsal process, localize to a set of structures with Broca's area as the principally activated brain region.

These results indicate that cognitive subtraction, the logic underlying much of cognitive neuropsychological investigation, is supported at least in this case^6^. All other components of the phonological loop system appear to be normally activated and normally located in JB. Very little re-organization of the anatomy sub-serving phonological loop skills seems to have taken place in patient JB in the 40 years since her brain damage.

These findings clearly allow us to reject the hypothesis that all properties of the phonological loop arise only from distributed activity involving all anatomical components, with no functional specialization in any of the relevant peri-sylvian areas. Rather, as suggested by early and more recent lesion studies (Risse et al., 1984; Vallar et al., 1997) and by functional imaging data in normal subjects (Paulesu et al., 1993; Demonet et al., 1994; Salmon et al., 1996), our data support the notion of a dissociation between anterior and posterior perisylvian areas as far as the cognitive architecture of the phonological loop is concerned. These findings also weaken the case for cognitive models of the phonological loop that do not assume the existence of at least two components, such as that of Hulme and Tordoff (1989).

The preserved activation in a rhyming task of components of the phonological loop such as Broca's area, which are anatomically normal, suggests that the components of the phonological loop neural system exhibit a degree of functional independence. A lack of connectivity with a destroyed key region *does not affect* the functional properties in the kind of phonological processing assessed in this experiment.

There are two caveats to the above arguments.

#### First caveat

The brain damage in patient JB was not restricted to the left temporo-parietal junction (Paulesu et al., 1993, 1995, 1996b; Demonet et al., 1994), but extends quite deeply into the region where the superior temporal sulcus and middle temporal gyrus normally are found. Indeed, the lesion invades cortical fields not necessarily involved in phonological short-term memory, as the severe aphasia at onset demonstrated. These additional areas in the middle temporal gyrus and superior-temporal sulcus are likely to be lexical-semantic in function (Howard et al., 1992; Price, 2000; Binder et al., 2009). Other language skills, defective when JB was aphasic, presumably must be subserved by other cortical regions given her normal performance in all language tasks except verbal short-term memory, in which there has been no recovery. JB's lesion does not however extend to the putative more posterior parietal localization for the phonological input buffer derived from the verbal working memory experiments of Smith et al. (1995, 1996) and Awh et al. (1996). See Figure 3 for a meta-analysis with reference to JB's lesion and her activations during a rhyming task^7^.

Based on observations in normal subjects (Paulesu et al., 1993), using PET/MRI co-registration (Paulesu et al., 1995), and on the basis of a meta-analysis of cases with acquired brain damage, (Shallice and Vallar, 1990; Vallar et al., 1997) the area crucial for the STMS appears to be in the left temporo-parietal junction in a cortical field area that many call *planum temporale* (Geshwind and Levitsky, 1968). The planum temporale is essentially part of Wernicke's area with an extension into parietal cortex, and it is to the parietal portion of the planum where our and others' findings localize the phonological store (Paulesu et al., 1993, 1995; Demonet et al., 1994; Salmon et al., 1996)^8^.

Galaburda and Sanides (1980) called this region area TPT, namely the temporo-parietal association cortex around the caudal end of the Sylvian fissure. Area TPT has a homolog in the monkey that has been studied by single cell recording (Leinonen et al., 1980). The monkey TPT has a predominant proportion of acoustic neurons (54%) that fire with complex auditory stimuli, including human consonant sounds (Leinonen et al., 1980). JB's lesion is larger than area TPT. However, it definitely incorporates the location where we place the phonological store in normal subjects.

The localization of JB's lesion is, though, entirely compatible with the location of the proposed auditory-motor speech interface claimed by Buchsbaum and D'Esposito (2008) namely the left sylvian-parietal-temporal area in the most posterior part of the planum temporale and the left posterior superior temporal region favored by Leff et al. (2009). These overlapping areas are clearly damaged in JB's brain (see Figure 3). The theoretical account of Buchsbaum and D'Esposito (2008), however, places the primary phonological buffer within the speech production system (see also Page et al., 2007) and explains JB's difficulty as an inability to access this store with the speech production system itself being intact. However, the more anterior parts of the speech system are intact in this study. Such a view, though, would need to explain what phonological trace is used in our very considerable capacity for veridical surface structure in immediate sentence recall (Jarvella, 1971, 1979; Glanzer et al., 1981) and why JB had lost the ability to reproduce surface structure but could perform much better at recall of gist (Shallice and Butterworth, 1977; Butterworth et al., 1990).

Current knowledge about the anatomical connectivity between different human cortical areas has grown considerably thanks to diffusion tensor imaging (DTI) techniques. It is reasonable to assume that in normal subjects, the brain areas damaged in JB are anatomically connected with anterior language areas such as Broca's area and with contralateral temporo-parietal areas. This idea is supported by evidence provided by DTI MRI tractography (Catani and Mesulam, 2008; Catani and Thiebaut de Schotten, 2008). We therefore discuss (1) whether JB's symptoms could be framed, anatomically, as the result of a disconnection syndrome, in line with some early proposals (Kleist, 1916; Kinsbourne, 1972) and (2) the significance of the preserved activation in Broca's area and contralateral cortices.

A direct connection between Wernicke's area and Broca's area was postulated by Meynert (1865) who suggested the existence of an *arcuate fasciculus* and this is largely confirmed by modern tractography (Catani et al., 2002; see also Catani and Mesulam, 2008 for a review). The functional lesion in JB definitively extends into white matter underlying the supramarginal gyrus. Could JB's deficit arise therefore from a disconnecting lesion in the white matter? It is difficult to rule out this hypothesis completely, although neuropsychological assessment of the patient suggests that a disconnection syndrome is an unlikely explanation at least in cognitive terms (see Shallice, 1988, p. 50–54, for a discussion of neuropsychological evidence against a disconnection hypothesis for the interpretation of case JB and similar patients).

Classical schemes from aphasiology also predict that lesions to an arcuate fasciculus should cause difficulties with word repetition as described in conduction aphasia, so that the deficit of patients like JB could be considered a sub-type of *conduction* aphasia. To date, however, there is very little evidence that an isolated white matter lesion can produce the syndrome of conduction aphasia. According to Damasio (1992), “the condition is related to damage in area 40 in the left cerebral hemisphere (supramarginal gyrus), with and without extension to the white matter beneath the insula…., (or to damage of)…left primary auditory cortices (areas 41 and 42), the insula and the underlying white matter.” It has also been found that when the lesions causing conduction aphasia are restricted to the arcuate fasciculus, the clinical picture is very mild (Poncet et al., 1987). To differentiate JB from such classical conduction aphasia patients it is important to recall that JB can reproduce single words and sentences very well. She had a *repetition deficit* typical of classical conduction aphasia, not the *reproduction deficit*, to use the terminology of Shallice and Warrington (1977).

#### Second caveat

This constitutes the most challenging aspect of our PET findings. There is a discrepancy in the involvement of mirror peri-sylvian regions of the right hemisphere. In particular, could observations of such activation in the present case where the left-sided mirror regions are destroyed make one reconsider potential re-lateralization of function? Do these activations mean that JB is actually performing the rhyming task with a different system? Is this a sign of functional re-organization of the phonological loop after brain damage? Do these findings invalidate the link between normal and abnormal models of the phonological loop?

At the time of writing, no specific function has been firmly attributed to right hemispheric peri-sylvian areas homolog to the phonological loop ones. It should be noted that, the activation of Broca's area is more prominent in JB on the left both in terms of spatial extent and of statistical significance suggesting that no general re-lateralization of language to the right hemisphere has occurred^9^. However, a verbal span of the order of 3 has been attributed to an isolated right hemisphere in certain split-brain patients (Zaidel, 1986), who nevertheless may have undergone some re-lateralization of function following early lesions. PET studies, analyzed on a group basis, suggest that the right peri-sylvian areas may not be crucial to perform rhyming tasks in normal subjects; however, such activity has been observed in normal subjects during verbal span tasks (Paulesu et al., 1993), although it is much smaller in spatial extent and significance than that on the left. The current rhyming task challenged JB at the limit of her pathological span (see Table 1). This suggests the possibility that in normal subjects right hemisphere systems concerned with phonological processing provide a qualitatively equivalent but quantitatively small contribution to that of the left hemisphere, so that in normal subjects the contribution is of little value but in some neurological patients it may be of value to their performance which is overall much reduced (a similar argument concerning the role of the right hemisphere is discussed with respect to models of acquired dyslexia by Plaut and Shallice, 1993).

Thus, right hemisphere activation in JB may reflect the use of a “normal,” quantitatively low, right hemisphere phonological loop for any minor span aspects of the task. This would be consistent with old accounts of conduction aphasia (Kleist, 1916; Kinsbourne, 1972). These, however, do not affect inferences to the organization of the normal cognitive system (Shallice, 1988).

### Recovery from aphasia: why phonological-short term memory does not recover in patients like JB?

If our interpretation is correct that JB performs the rhyming task essentially with the remains of a normal phonological short-term memory system, it follows that this system has functionally and anatomically little chance for compensation, unlike other skills/systems that were defective at the onset of the disease but have seen considerable recovery. In this respect JB's enduring deficit in phonological short-term memory skills after stabilization of brain damage is no exception as shown by long-term studies in other similar patients, like patient PV (Vallar and Baddeley, 1984). If this position is correct, one would not expect to find a patient with a lesion in the critical anatomical area who regains phonological short-term memory abilities while other aphasic problems remain.

The reason why phonological short-term memory shows little if any recovery is for the moment a matter of speculation. A comparison with the normal process of second language learning in adulthood may help understand the lack of compensation of a span deficit over many years in patients like JB. Phonological competence for a second language is reduced the later one learns it (Cutler et al., 1986, 1989, 1992). This is as if, after a certain age, the phonological system “crystallizes” around the phonological/articulatory representations of the first or dominant language. This is evidence of limited plasticity of the phonological articulatory system in normal adulthood, even for normal linguistic processes such as learning a second language. The analogy between limited phonological competence in second language acquisition by adults and the lack of verbal span recovery in patients like JB is strengthened if one recalls that similar patients become virtually unable to learn new words in a second language (Baddeley et al., 1988), while all other aspects of episodic (verbal and non-verbal) long-term memory can remain intact.

## Limitations of the PET experiments and further fMRI data on elderly subjects

Recent evidence is showing that elderly subjects may perform a given cognitive task at the same level as younger subjects, while showing different patterns of brain activation (Cabeza, 2002; Dolcos et al., 2002). In particular, elderly subjects may show broader and more bilaterally distributed activations, particularly in the frontal lobe (Cabeza, 2002; Dolcos et al., 2002; Cabeza et al., 2004). JB showed great similarity with normal controls except for the right-sided TPT and right “Broca's” regions. However, as the control group of the PET experiment was not matched for JB's age, to better interpret these differences we performed a further experiment using fMRI in age matched normal volunteers. JB was no longer alive at the time of this further experiment. We envisaged three possible scenarios: (a) most elderly subjects activate the mirror regions of left area TPT and Broca's area (as in a group analysis) suggesting that JB's right sided pattern is consistent with what is seen in a sample of subjects representative of her age; (b) no such activation is present in any of the normal elderly controls leaving open the possibility that JB was using a minor mirror phonological loop together with left Broca's area as a compensation for her left sided brain damage^10^; (c) activation in right sided regions seen in JB is present in some elderly controls suggesting that such activation may be a trait in some subjects thus making JB less of an exception.

### Methods

#### Subjects

The group included five male and nine female right handed subjects (mean age = 60; s.d. = 6.38). All subjects had no medical history of neurological disorders. All participants gave their informed written consent to take part in the study; the study was approved by the Ethics Committee of the Niguarda Ca' Granda Hospital of Milan.

#### Methods

During fMRI scanning, the same stimulation procedures (a visual rhyming task and a visual similarity detection on false font) were adopted as for the PET scans, the difference being that each PET scan corresponded to a 30″ block of 10 fMRI scans.

#### fMRI acquisition data

MRI scans were performed on a 1.5 T Marconi-Philips Infinion Scanner, using an Echo Planar Imaging (EPI) gradient echo sequence (Flip angle 90° TE = 60 ms, TR = 3 s, FOV = 240 × 240, matrix = 64 × 64). The selected volume was made of 26 contiguous transverse images (thickness = 5 mm; gap = 0 mm), acquired every 3.05 s. The scans were collected parallel to the AC-PC plane.

The fMRI experiment involved 120 fMRI scans collected in alternating blocks of 10 scans of baseline (shape similarity judgment) and experimental (visual rhyming on letter names) task.

#### fMRI data analysis

After a standard pre-processing, high-pass filtering and proportional scaling, conditions were modeled in a block-design and condition-specific effects were estimated using SPM12. The BOLD signal was convolved with a canonic hemodynamic response function. These analyses generated for each subject contrast images containing statistical information about fMRI signal changes observed at a given statistical threshold. These contrast images were then entered into a one-sample *t*-test analysis for group inference (Friston et al., 1999). The effect was thresholded at *p* < 0.001 uncorrected at voxel-level and at *p* < 0.05 FWE-corrected at cluster-level (cluster size = 350).

Single subject analyses were also performed for the right mirror regions of area TPT and Broca's area. Here we used the right activation observed in JB as an inclusive mask

### Results

#### Behavioral results during fMRI

Normal controls performed on average 98% correct for the rhyming task and 98% correct for the shape similarity task. Table 4 reports the individual performances and d-prime values.

**Table 4:** **The demographic data, the individual performances, and the D prime values**.

**ID**	**Age**	**Educational level**	**Gender**	**Shape similarity task**				**Letter rhyming task**			
			**(1 = male; 2 = female)**	**Hits**	**Correct rejects**	**% correct response**	**D prime**	**Hits**	**Correct rejects**	**% correct response**	**D prime**
1	67	13	1	26	26	100	2.67	28	53	100	3.98
2	57	13	1	26	64	100	6.57	28	62	100	6.58
3	69	15	1	26	64	100	6.57	28	62	100	6.58
4	64	12	2	24	64	92	5.09	27	60	96	3.64
5	53	18	2	25	62	96	3.63	28	62	100	6.58
6	58	18	1	26	49	100	3.63	27	61	96	3.94
7	68	18	2	23	63	88	3.35	24	61	86	3.21
8	60	13	2	26	64	100	6.57	28	62	100	6.58
9	56	13	2	26	64	100	6.57	28	60	100	4.77
10	50	18	2	25	64	96	5.43	26	62	93	5.12
11	68	13	1	26	64	100	6.57	28	62	100	6.58
12	64	8	2	26	60	100	4.44	28	61	100	5.07
13	55	5	2	26	64	100	6.57	28	59	100	4.59
14	54	17	2	26	62	100	4.77	28	60	100	4.77
Mean	60	–	–	–	–	98	5	–	–	98	5
S.d.	6.38	–	–	–	–	3.62	1.44	–	–	4.14	1.23

#### fMRI results

**Figure 4:**
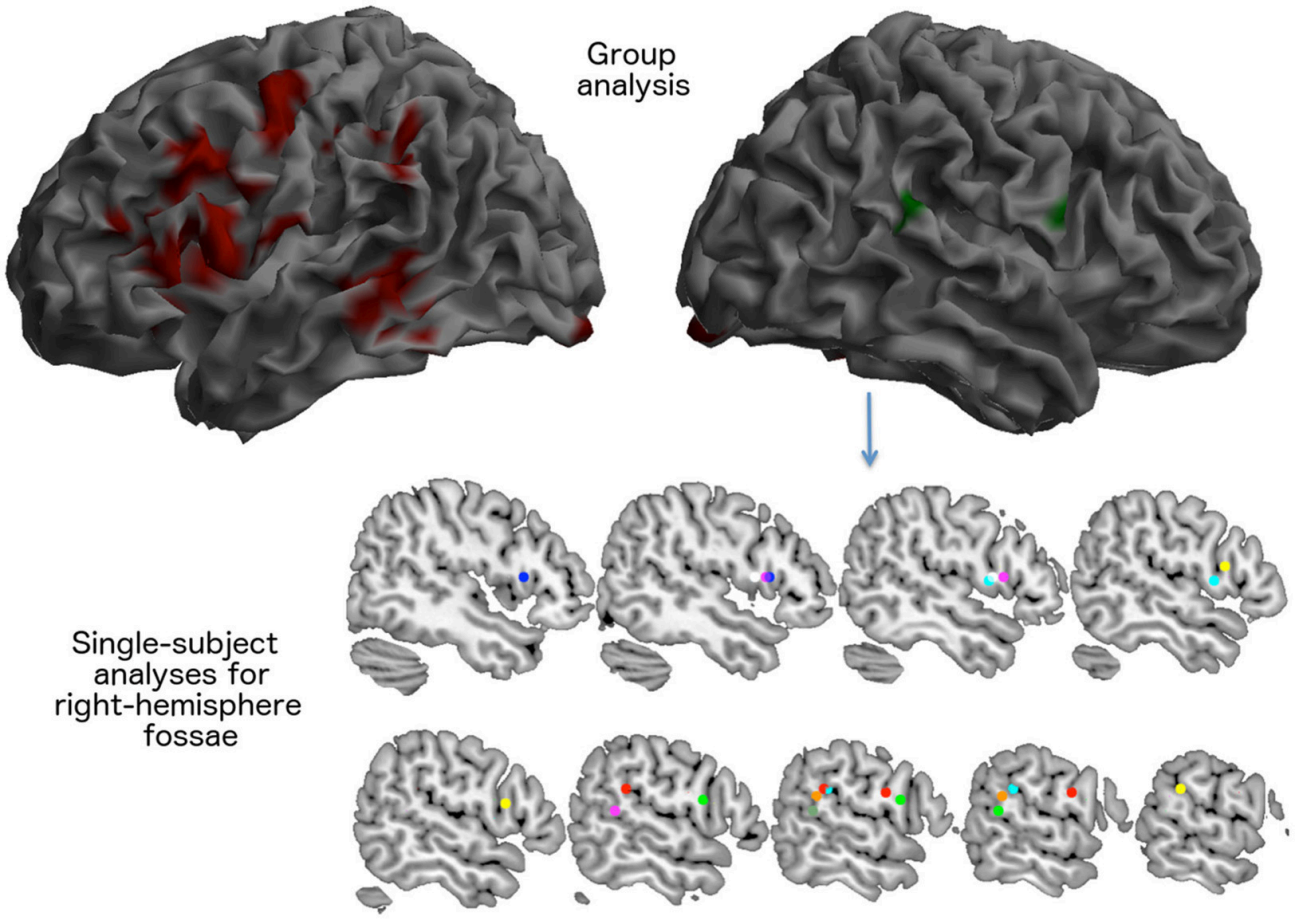
**Brain areas activated for the rhyming tasks as seen in 14 elderly control participants during the fMRI scan described in experiment 2**. The visual rhyming task was the same used with patient JB. On the left, the group effect (areas in red) that survived a *p* < 0.05 FWE cluster level corrected threshold (primary voxel level threshold: *p* < 0.001 uncorrected). The right hemisphere rendering on the right illustrates the average location of right hemispheric activations. Right sided activations were observed only in eight subjects (areas in green). In the lower part of the figure, the location of the local maxima of the activations seen in the right inferior frontal cortex and in the right temporo-parietal junction are reported for each subject.

In normal elderly subjects, performance of the rhyming tasks was associated with a large activation of the left inferior frontal/premotor region, of the left temporo-parietal junction included the supramarginal gyrus, much as it is seen in normal young controls during phonological short-term memory tasks. Compared with previous PET data results (Paulesu et al., 1993, 1996b), there were also activations in the temporal lobe, in the calcarine cortex, in the basal nuclei in the hippocampus and in the thalamus. There was also a left activation of the cerebellum (see Table 5 and Figure 4).

**Table 5:** **Brain areas activated during rhyming task in elderly controls**.

**Brain regions**	**Left hemisphere**				**Right hemisphere**			
	***x***	***y***	***z***	***Z* score**	***x***	***y***	***z***	***Z* score**
Inf. frontal orb. gyrus	−28	24	−12	4.1	–	–	–	–
	–36	24	–4	3.5	–	–	–	–
Inf. frontal tri. gyrus	–38	16	32	4.6	–	–	–	–
	–44	26	10	4.4	–	–	–	–
Inf. frontal op. gyrus	–40	20	34	4.4	–	–	–	–
Rolandic opercular gyrus	–56	6	10	4.1	–	–	–	–
	–62	–8	12	3.6	–	–	–	–
Precentral gyrus	–42	10	34	4.68	–	–	–	–
	–48	8	32	4.55	–	–	–	–
Postcentral gyrus	–42	–22	40	4.0	–	–	–	–
	–38	–24	42	3.8	–	–	–	–
Insula	–44	18	2	6.0	–	–	–	–
	–34	14	6	4.6	–	–	–	–
Supramarginal gyrus	–58	–38	38	3.8	–	–	–	–
	–60	–44	28	3.5	–	–	–	–
Mid. temporal gyrus	–48	–46	2	4.3	–	–	–	–
	–58	–32	–6	4.2	–	–	–	–
Inf. temporal gyrus	–58	–50	–22	3.7	–	–	–	–
	–56	–44	–18	3.3	–	–	–	–
Inf. occipital gyrus	–20	–94	–10	3.1	–	–	–	–
Calcarine fissure	–8	–102	–14	3.5	2	–92	–12	3.5
	–24	–98	–14	3.5	–	–	–	–
Lingual gyrus	–14	–102	–16	3.7	–	–	–	–
Cerebellum	–42	–82	–22	3.9	–	–	–	–
	–40	–86	–20	3.8	–	–	–	–
Caudate	–16	–2	18	3.9	–	–	–	–
Putamen	–26	–2	18	3.9	–	–	–	–
Pallidum	–20	–2	16	4.0	–	–	–	–
Thalamus	–12	–10	6	4.8	–	–	–	–
	–8	–4	–2	4.0	–	–	–	–
Hippocampus	–26	–24	–14	4.6	–	–	–	–
	–34	–22	–14	4.2	–	–	–	–

*The effect was thresholded at p < 0.001 (uncorrected) and the cluster-size level was set at 350 voxels (p < 0.05 FWE-corrected)*.

Notably, at a group level, there was no significant right activation, neither of right Broca's region, nor of the right TPT.

However, the single subject analyses showed that right “Broca's” area was activated in seven elderly normal subjects, while the right TPT was active in six (see Table 6 and Figure 4). Of these subjects, five had activation in both regions.

**Table 6:** **Local maxima of the single-subject activations seen in the right inferior frontal cortex and in the right temporo-parietal junction**.

**ID Subject**	**Temporo-parietal junction**				**Inferior frontal gyrus**			
	***x***	***y***	***z***	***p***	***x***	***y***	***z***	***p***
1	–	–	–	–	–	–	–	–
2	58	−36	24	<0.001	60	−2	22	<0.001
3	–	–	–	–	–	–	–	–
4	–	–	–	–	48	16	10	<0.001
5	60	−42	12	<0.001	58	6	18	0.001
6	60	−40	20	0.003	–	–	–	–
7	64	−34	24	0.005	54	12	16	0.004
8	–	–	–	–		–	–	–
9	–	–	–	–		–	–	–
10	–	–	–	–	50	8	10	0.003
11	–	–	–	–	–	–	–	–
12	60	−34	24	0.001	52	6	8	0.001
13	58	−42	12	0.005	50	14	10	0.001
14	–	–	–	–	–	–	–	–

### Discussion of experiment two

The implications of the fMRI results are simple and straightforward. They confirm the involvement of left peri-sylvian regions in aspects of phonological processing implied by a simple rhyming task for single letter names. Activation of the left temporo-parietal cortex, the region that was damaged in JB and that we associated with the phonological buffer in previous PET studies, suggests that this region may also contribute to low-level phonological tasks, although less prominently, as demonstrated by the previous quantitative comparison with a higher load short-term memory task (Paulesu et al., 1993, 1996b). Indeed, a sub-threshold activation of this region was present in the young normal controls studied with PET (cf. the bar-graph of the rCBF increase of this region in Figure 2 of Paulesu et al., 1993). The use of a more sensitive technique, such as fMRI, allowed us to observe a significant hemodynamic response in this region.

The more relevant finding of the fMRI experiment, however, is the observation that the right-sided peri-sylvian region activates with the rhyming task in a number of healthy elderly subjects too. Accordingly, the right-sided activations seen in JB are not necessarily a sign of reorganization.

## Conclusions

As a consequence of her selective cognitive and anatomical deficits, the findings in patient JB support the notion that the general principle of functional separation at a cognitive level can be observed at the functional anatomical level as well, at least for some systems like the phonological loop.

This conclusion would have been hard to draw by studying normal subjects only. A combination of observations in normal subjects and in neuropsychological patients seems vital to validate cognitive and neurophysiological models that imply a certain degree of modular organization.

Our observations do not necessarily exclude the value of the interaction between different components of distributed systems in generating complex aspects of behavior and of course, it may also be the case that these principles are only applicable to a subset of cognitive operations; for example, semantic operations and other executive functions may differ, but that remains to be demonstrated.

However, at a specific conceptual grain, the idea of individual sub-systems retains explanatory power. Moreover, our conclusions support one of the most basic assumptions of cognitive neuropsychology, namely that the subtraction logic works, at least in the domain of phonological short-term memory.

## Ethics statement

This studies were carried out in accordance with the recommendations of the Hammersmith Hospital and of the Niguarda Ca' Granda Hospital Ethics Committees. All subjects gave written informed consent in accordance with the Declaration of Helsinki.

## Author contributions

EP and TS: Contributions to the conception and design of the work; acquisition and analysis of the first experiment, interpretation of data for the work, Drafting the work. LD: Contributions to the conception and design of the second experiment; acquisition and analysis of the second experiment. MS: acquisition and analysis of the second fMRI experiment. RF and CF: Contributions to the conception and design of the work; Drafting the work.

### Conflict of interest statement

The authors declare that the research was conducted in the absence of any commercial or financial relationships that could be construed as a potential conflict of interest.

## Footnotes

^1^A major discrepant finding in this respect is the study of Leff et al. (2009). They used a VBM-style analysis of 210 stroke patients who had undertaken a comprehensive aphasia battery, poorer performance on span was found in a large region of the left hemisphere. They then take out the effects of five regressors—auditory word reproduction, auditory non-word repetition, verbal fluency, verbal fluency, and stroke volume. Now the digit span deficit region is limited to a small posterior superior temporal region (*x* = −66; *y* = −32, *z* = 4). However, it could be argued that the study used one regressor too many; auditory non-word repetition may well-load on input phonological buffer capacity (but see Acheson et al., 2011). Yet another patient series, using VLSM implicates both the superior temporal and inferior parietal regions while individual word repetition implicates only the temporal portion (Baldo et al., 2013). A more generic deficit in the performance of the digit span, compared for example with the performance of the Token test, once mapped anatomically on a population level with voxel-based symptom lesion mapping, again correlates with the involvement of the left temporo-parietal junction (see for example, Koenigs et al., 2011).

^2^Lesions identified with rCBF PET scans may not correspond fully to structural lesions; in chronic stroke, the area of full structural damage has blood flow close to zero, surrounded by areas of reduced blood flow. Accordingly, in the paper the wording “brain lesion” for JB implies a combination of structural and/or functional damage.

^3^The lesion area was masked and not included in stereotactic normalization so that it did not affect the transformations of healthy brain tissue at this stage of pre-processing.

^4^There is recent imaging evidence suggesting a number of verbal short-term memory sub-regions at the junction between the superior temporal region and the supramarginal gyrus or in left temporal cortex: these show response patterns that, for the time being, one would find difficult to correlate with classical phonological loop phenomenology. This level of resolution we consider below the grain size of description of primary effects of phonological short-term memory, like for example, the capacity of the store, short-term memory for identity or order, the phonological similarity effect or the word length effect and their interactions with the modality of stimuli presentation.

^5^In describing a localized activation effect, ideally one would like to use stereotactic coordinates to preserve the anatomical precision in any statement. Of course, this is impossible in narrative. In the original description of the anatomy of the phonological loop, Paulesu et al. (1993) used supramarginal gyrus and Brodmann's area 40 in alternation, as synonyms, mostly for stylistic reasons. Given the large anatomical extension of BA 40 and of the supramarginal gyrus, this has generated considerable confusion inviting some to consider their findings in parietal lobe as replications of Paulesu et al. (1993), in spite of an anatomical distance in stereotactic space in their findings of more than 2 cm (sic!) (Smith et al., 1995, 1996; Awh et al., 1996). If this seemed acceptable in the early 90s, it is now below the standards of anatomical accuracy, as pointed out by Buchsbaum and D'Esposito (2008). See Figure 3 for an anatomical meta-analysis of “phonological store” findings and its comparison with JB's lesion. Accordingly, we avoid this terminology here by using wordings that better reflect the stereotactic coordinates of Paulesu et al. (1993) original findings.

^6^These observations are somewhat in conflict with the rationale of the position held by Price and Friston (2002). They argued that “there may be more than one neural system for any given cognitive function with each system sufficient for intact performance. This “degeneracy” underlies individual variability; provides insights into the mechanisms sustaining recovery.” The implication of this position is that for any given cognitive function, the behavioral contribution of a minor subsidiary neural system is masked by the primary neural system, and revealed only by an acquired brain lesion. As a consequence, residual performance may not be interpretable in terms of the operations of a cognitively modular subcomponent of the normal system, rather by the operation of the minor system, which is not used in normal function. Normal and neuropsychological evidence would relate to essentially different processing systems and the data in one domain would provide little insight to the other. In particular neuropsychological data would not speak directly to the organization of the primary cognitive systems involved in the function. The examples given by Price and her colleagues both relate to patients who were held to be essentially normal on a particular task. However, they do not provide a discussion of the theoretical relevance to patients who do show a selective deficit—the standard situation in cognitive neuropsychology.

^7^Becker et al. (1999) and Buchsbaum and D'Esposito (2008) have pointed out that there are studies, particularly those using the so-called n-back procedure, which localize the phonological input buffer much more posteriorly and dorsally in the left parietal cortex (e.g., Smith et al., 1996, 1995; Awh et al., 1996). However, Buchsbaum and D'Esposito (2008) and Shallice and Cooper (2012) argue that the n-back task is not well-suited to localizing the phonological input buffer. The phonological storage requirements are much less than for span tasks. Moreover, Buchsbaum and D'Esposito (2008) have suggested that the more posterior parietal localization might relate to the shifting of attention involved in the n-back task rather than to the location of a buffer (see Figure 3).

^8^Ravizza et al. (2011) propose an alternative localization for the phonological buffer. They observed that the superior temporal gyrus, rather than the left temporo-parietal junction, was particularly active during encoding with a time related decay of the signal demonstrating, according to them, the expected profile of a store. While their methodology, was sufficiently different from earlier studies to justify different results (e.g., a percentage signal change against the beginning of each trial was used as a dependent variable rather than the difference against time matched activity for non-phonological material), subjects actively rehearsed the stimuli while the signal in the superior temporal gyrus decayed; signal decay and active rehearsal are not readily compatible in the canonical phonological loop model as rehearsal should counterbalance the spontaneous decay of memory traces from the store.

^9^The statement on the region by hemisphere difference during the rhyming task in Broca's area for patient JB holds true when tested formally in a statistical analysis by looking at task by hemisphere interactions: *p* < 0.001.

^10^Of course there is no question on the left-sided hemispheric dominance for language in JB given the severe aphasia at onset and the enduring phonological short-term memory deficit.
