# Supplementary material for: Anatomical Modularity of Verbal Working Memory? Functional Anatomical Evidence from a Famous Patient with Short-Term Memory Deficits
Source: Front Hum Neurosci. 2017 May 17;11:231. doi: 10.3389/fnhum.2017.00231 (PMC5434108; doi:10.3389/fnhum.2017.00231)

**Supplementary materials**

Further analysis on a group of five dyslexics described in Paulesu et al (1996b) and studied with the same methodology as in Paulesu et al. (1993). The data confirm the basic observations of Paulesu et al. (1993), based on normal subjects, on the brain region more active for the phonological short-term memory task than for a rhyming task using visually presented single letter names, after subtraction of the rCBF response for matched visual tasks. The activation was characterized as an interaction effect in a 2 x 2 design, with two phonological tasks (with and without short-term memory demands, a span task and a rhyming task) and two visual analysis/visual short-term memory tasks. The brain region was significantly more active for the phonological task when short-term memory demands were present. The stereotactic coordinates of this effect and the exact anatomical location are descried below.

|  |  | | | | | | | | |
| --- | --- | --- | --- | --- | --- | --- | --- | --- | --- |
|  | **Left hemisphere** | | | |  | **Right hemisphere** | | | |
|  | *x* | *y* | *z* | *Z score* |  | *x* | *y* | *z* | *Z score* |
| **Brain regions** |  |  |  |  |  |  |  |  |  |
|  |  |  |  |  |  |  |  |  |  |
| Supramarginal gyrus | -56 | -28 | 26 | 4.0 |  | - | - | - | - |
|  | -54 | -26 | 20 | 3.6 |  | - | - | - | - |
|  | -58 | -42 | 28 | 3.4 |  | - | - | - | - |
| Sup. Temporal gyrus | -66 | -36 | 20 | 4.7 |  | - | - | - | - |
|  | -58 | -38 | 22 | 4.1 |  | - | - | - | - |
|  | -64 | -42 | 22 | 4.0 |  | - | - | - | - |


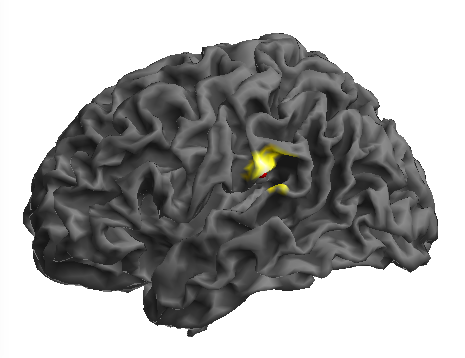

Supplement: Supplementary file 1 [file DataSheet1.DOCX]
